# Editorial: Advance in translational research of preterm birth and related pregnancy

**DOI:** 10.3389/fphar.2022.1047649

**Published:** 2022-11-03

**Authors:** Han Xie, Hao Ying

**Affiliations:** ^1^ Department of Obstetrics, Shanghai First Maternity and Infant Hospital, Tongji University School of Medicine, Shanghai, China; ^2^ Shanghai Key Laboratory of Maternal Fetal Medicine, School of Medicine, Shanghai Institute of Maternal-Fetal Medicine and Gynecologic Oncology, School of Medicine, Tongji University, Shanghai, China

**Keywords:** inflammation, infection, pathogenesis, preterm birth, therapeutics, translational research

Spontaneous preterm delivery accounts for approximately 8% of all live births worldwide (GBD 2016 Causes of Death Collaborators., 2017), which is the leading cause of perinatal mortality and the second major cause of death in children under 5 years old. Survivals of preterm birth are often faced with short- and long-term morbidity (Slomski, 2021). Adverse environmental exposure and changes in fertility policies may lead to a gradual increase in the premature birth rate (Liu et al.,2019; Deng et al., 2021). Infection-induced inflammation is thought to be the primary cause of rupture of membranes and uterine contraction which consequently results in preterm birth (Kim et al., 2015; Bian et al., 2021; Xu et al., 2021; Xie and Ying, 2020; Pan et al., 2020). However, due to the lack of sufficient understanding of the underlying mechanism, current diagnosis and treatment are often based on clinical symptoms, which sometimes yields inconsistent outcomes.

With the emergence of new technologies such as multi-omics analysis (Wang et al., 2022), searching for novel marker genes and signaling pathways related to premature delivery has become a research hotspot (Wang et al., 2018). As a result, a number of novel and pivotal signalers have been revealed, which may be of potential value for the determination of gestational age and prediction of delivery time. Metabolites and humoral factors derived from the fetus *per se* or fetal appendage appear to be particularly appealing (Chen et al., 2020; Liang et al., 2020; Wang et al., 2018). These findings offer deep insight into the mechanism of preterm birth and expand new visions for the prediction of preterm birth.

While uterine contraction inhibitors and antibiotics are important drugs for the treatment of preterm labor (Sharp and Alfirevic, 2014), the effect of hormones on promoting fetal lung maturation in premature infants has been verified over a wider range (WHO ACTION Trials Collaborators et al., 2020). Besides, the protective effect of aspirin or progestogens for preventing preterm birth was also found (EPPPIC Group, 2021; Hoffman et al., 2020). Faced with the complex mechanism and heterogeneity of clinical manifestations of spontaneous preterm birth, further optimization and clarification of clinical treatment remain highly warranted, which requires the translational investigation of the mechanism underlying preterm birth.

Therefore, this Research Topic aims to provide the readers with research evidence of effective treatment and novel mechanisms of spontaneous preterm birth, with emphasis on the inflammation of maternal and fetal tissues. Through the hard work of contributors, reviewers, and editors, a total of 9 articles were eventually accepted for publication, including 3 reviews and 7 original studies.

In terms of the pathogenesis of preterm birth, especially inflammation and infection, Saito Reis et al. demonstrated that fetal DNA related to genders, fragment size, and methylation status might cause inflammation in the fetal membranes *via* increased activation of NF-κB, MMP activity, and cytokine secretion, which broadens the theory of the role of fetus in the initiation of labor. A retrospective cohort study of singleton pregnancies with preterm premature rupture of membranes (PPROM) by Matulova et al. investigated the correlation between acute inflammation of the amnion and fetus birth weight, and their findings further supported that the severity of acute inflammation of the amnion may alter the growth of the fetus. Stranik et al. utilized ultrasound-guided transabdominal intra-amniotic administration of a triggering agent to establish a rat model of intra-amniotic inflammation, and they characterized that the concentration of interleukin (IL)-6 in the amniotic fluid may be of critical value to study intra-amniotic inflammatory complications and precisely mimic different specific clinical scenarios. Kacerovsky et al. analyzed 217 women’s cervical secretions and amniotic fluid and found that the presence of intra-amniotic infection, sterile intra-amniotic inflammation, or colonization of the amniotic fluid was associated with a higher prevalence and/or load of *Ureaplasma* spp. DNA in the cervical fluid than the absence of intra-amniotic complications in PPROM at <34 weeks, which confirmed the important role of vaginal microbiota in preterm birth-related diseases and its significance in clinical diagnosis and treatment (Keelan et al., 2015). Meanwhile, in terms of antibiotic treatment, there was consistent inconsistency in outcome selection and reporting in studies about antibiotics in PROM. Therefore, Liu et al. formed an initial core outcome set for antibiotics in PROM through a systematic review and semi-structured interview, which could improve the research quality of PROM and provide a reference for research on infection in pregnant women.

In addition, the mechanism of other related treatments has also attracted our attention. Yin et al. explored the feasibility of measuring the uterine and peripheral artery diameters after the administration of different doses of ephedrine using CT and showed that the peripheral artery contracts under the action of ephedrine, whereas the common clinical dose of ephedrine has no significant effect on the diameter of the uterine artery, which will provide a reference for the scientific and rational use of ephedrine in the clinic and, ultimately, improve the safety of patients and fetuses undergoing cesarean section required for premature delivery. The review by Devvanshi et al. highlighted the prospects of exosomes as therapeutic tools and early diagnostic markers at the immune level in adverse pregnancies such as PPROMs. A systematic review and meta-analysis of randomized evidence by Xinyu et al. indicated that prophylactic use of motherwort injection may reduce the risk of uterine hemorrhage in women after abortion, which suggested the potential effect of traditional Chinese medicine. Hsu et al. demonstrated that pregnancy stress was significantly lower in pregnant women who were receiving tocolytic treatment than in those who were receiving non-tocolytic treatment among both who used complementary medicine. This finding can be used as a reference for future studies on pregnant women’s health.

In conclusion, this Research Topic has provided new experimental data and updated reviews related to translational research of preterm birth and related pregnancy. These studies further advance our understanding of spontaneous preterm birth pathogenesis. The evidence gathered from this Research Topic is also expected to be translated into more accurate and effective clinical approaches to predict and treat preterm birth in the future.
